# Supplementary material for: Alcohol and e-cigarette damage alveolar-epithelial barrier by activation of P2X7r and provoke brain endothelial injury via extracellular vesicles
Source: Cell Commun Signal. 2024 Jan 15;22:39. doi: 10.1186/s12964-023-01461-1 (PMC10789007; doi:10.1186/s12964-023-01461-1)
Supplement: Supplementary file 1 — Additional file 1. [file 12964_2023_1461_MOESM1_ESM.docx]

Alcohol and e-cigarette damage alveolar-epithelial barrier by activation of P2X7r and provoke brain endothelial injury via extracellular vesicles

Naveen Mekala, Jayshil Trivedi, Priyanka Bhoj, Namdev Togre, Slava Rom, Uma Sriram, and Yuri Persidsky^*^.

Department of Pathology and Laboratory Medicine, Lewis Katz School of Medicine, Temple University, Philadelphia, PA 19140, USA.

**Figure S1: Effect of chronic ETH stimulation on hPAEpiC mitochondrial function regulating cell viability**. Confluent hPAEpiC were incubated overnight with 10mM, 50mM, 100mM and 200mM ETH conditioned media. MTT assay was carried out to measure mitochondrial dysfunction and viability (mitochondrial succinate dehydrogenase catalyzes the MTT to MTT-formazan which is an indication of cell viability). Lower ETH concentrations (10mM and 50mM) had no significant effect on cell viability >90% viable). 200mM ETH was cytotoxic, recording a 60% cell viability. With 85% viability, 100mM ETH treatment found to be optimal to study mitochondrial stress in hPAEpiC. One-way ANOVA was used for statistical analyses. *P ≤ 0.05, ***P ≤ 0.001, ****P ≤ 0.0001, and ns (not significant).
