# Supplementary material for: Alcohol and e-cigarette damage alveolar-epithelial barrier by activation of P2X7r and provoke brain endothelial injury via extracellular vesicles
Source: Cell Commun Signal. 2024 Jan 15;22:39. doi: 10.1186/s12964-023-01461-1 (PMC10789007; doi:10.1186/s12964-023-01461-1)
Supplement: Supplementary file 2 — Additional file 2. [file 12964_2023_1461_MOESM2_ESM.docx]

Alcohol and e-cigarette damage alveolar-epithelial barrier by activation of P2X7r and provoke brain endothelial injury via extracellular vesicles

Naveen Mekala, Jayshil Trivedi, Priyanka Bhoj, Namdev Togre, Slava Rom, Uma Sriram, and Yuri Persidsky^*^.

Department of Pathology and Laboratory Medicine, Lewis Katz School of Medicine, Temple University, Philadelphia, PA 19140, USA.

**Figure S2: Time dependent paracrine signaling/Ca2+ accumulation in hBMVECs treated with hPAEpiC-EVs.** Confluent hBMVECs were incubated with freshly isolated hPAEpiC-EVs (1:300). Cells were collected and snap frozen at 1 h intervals. hBMVECs were lysed in Ca^2+^ assay buffer and calcium assay kit from Abcam (Cat. No. ab102505, Cambridge, UK) was used to measure intracellular Ca^2+^ levels according to manufacturer’s instructions. Intracellular Ca^2+^ accumulation started to appear after 3 h, whereas Ca^2+^ levels peaked after 5 h. One-way ANOVA was used for statistical analyses, ***P* ≤ 0.01, ****P* ≤ 0.001 and ns (not significant) (n=3).
