# Supplementary material for: Alcohol and e-cigarette damage alveolar-epithelial barrier by activation of P2X7r and provoke brain endothelial injury via extracellular vesicles
Source: Cell Commun Signal. 2024 Jan 15;22:39. doi: 10.1186/s12964-023-01461-1 (PMC10789007; doi:10.1186/s12964-023-01461-1)
Supplement: Supplementary file 3 — Additional file 3. [file 12964_2023_1461_MOESM3_ESM.docx]

Supplementary File of Uncropped Western blots

IRE1alpha


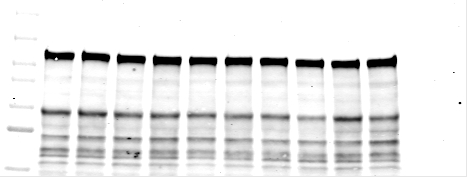


**IRE1⍺ (110kDA)**


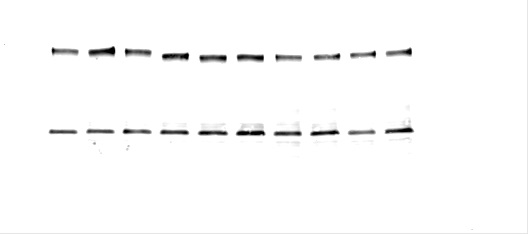


**IRE1⍺ (110kDA)**


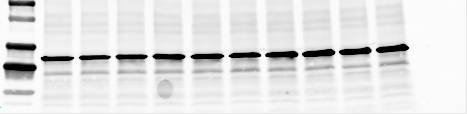


**β-actin (42kDA)**

blot scanned for longer durations, visually don’t show the differences in band intensities. When scanned for shorter periods we can see the visual changes. I have attached both images for your information.

pASK1


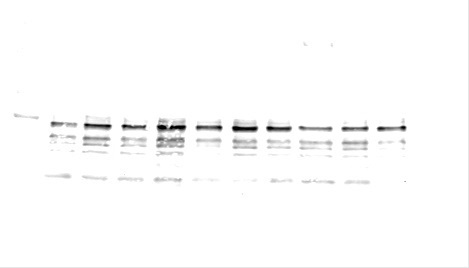


**β-actin (42kDA)**

**IRE1⍺ (110kDA)**


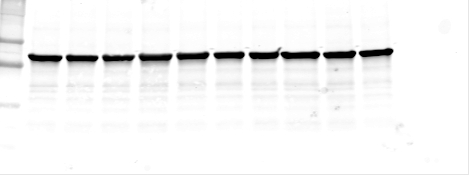


**pASK1 (155kD)**

**β-actin (42kDA)**

Bax Inhibitor-1 (BI-1
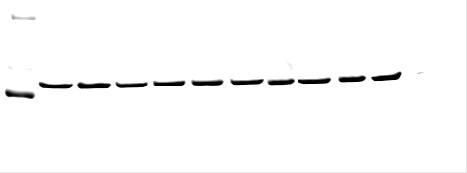

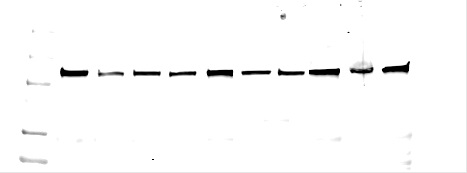
)

**BI-1 (25kDA)**

**β-actin (42kD)**

CD81 and CD21


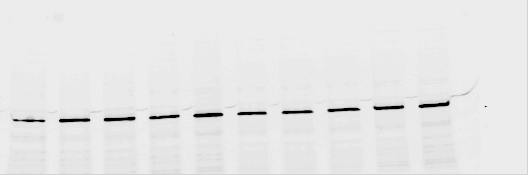

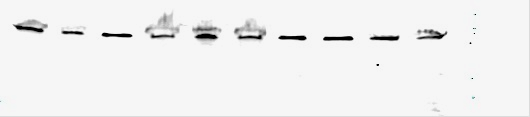


CD9 (26kDA)

CD81 (26kDA)

CD81 (26kDA)

CD9 (26kDA)
